# Paraquat Poisoning Associated With Daisley Barton Syndrome: A Case Report

**DOI:** 10.7759/cureus.19287

**Published:** 2021-11-05

**Authors:** Nidhi Kaeley, Hari Prasad, Ankita Kabi, Alok Raj, Archana Bairwa

**Affiliations:** 1 Emergency Medicine, All India Institute of Medical Sciences, Rishikesh, Rishikesh, IND; 2 Emergency Medicine (Anaesthesiology), All India Institute of Medical Sciences, Rishikesh, Rishikesh, IND

**Keywords:** case report, pesticides, pneumothorax, pneumomediastinum, paraquat

## Abstract

Pesticides include insecticides, herbicides, and rodenticides. Pesticide poisoning can be intentional, accidental, or occupational. Around 385 million cases of unintentional acute pesticide poisoning occur annually worldwide, with approximately 11,000 fatalities. Herbicides are used to kill weeds and can include chlorophenoxy compounds, bipyridyls, urea-substituted herbicides, organophosphates, and glyphosate. Paraquat is a bipyridyl nonselective contact herbicide with high mortality rates upon exposure in humans. Paraquat poisoning causes acute lung injury, rarely leading to pneumothorax and pneumomediastinum, referred to as Daisley Barton Syndrome. We report a case of a 22-year-old female from Uttarakhand, India, who accidentally ingested paraquat. She was initially asymptomatic, but later developed lung, liver, and kidney injuries as well as pneumomediastinum and pneumothorax.

## Introduction

Paraquat poisoning has been reported worldwide, but only a few cases have been reported in India [1]. Paraquat (1,1′-dimethyl-4,4′-dipyridylium) is a toxic, corrosive liquid with a strong odor [2]. After ingestion, it mainly enters the lungs, liver, and kidneys [3]. The principal affected organ is the lungs [1]. Besides local corrosive damage to the oral cavity and gastrointestinal mucosa, paraquat also causes metabolic acidosis, acute kidney and liver injury, pulmonary fibrosis, and acute respiratory distress [4]. Very rarely, it can cause spontaneous pneumothorax or pneumomediastinum [5]. Paraquat-induced acute lung injuries, namely pneumothorax and pneumomediastinum, are collectively referred to as Daisley Barton Syndrome [6]. We present a case of Daisley Barton Syndrome in a 22-year-old female after the ingestion of paraquat.

## Case presentation

A 22-year-old female with no known comorbidities or history of taking medications presented to the emergency department of a tertiary care institute in Uttarakhand after having ingested 100 mL paraquat-contaminated water from her farmyard. Upon admission, she reported a history of blue-colored vomiting and dizziness and was immediately taken to the local hospital. Gastric lavage was not performed because of the corrosive nature of the poison. The patient was admitted there and discharged after two days. On day four after ingestion, she started experiencing burning pain in her upper and lower limbs, which worsened at night and disturbed her sleep. The patient complained of dyspnea on exertion for one day, which was Modified Medical Research Council (MMRC) Grade II that worsened to Grade III. It was not associated with cough, hemoptysis, chest pain, or orthopnea. She also complained of yellowish discoloration of the eyes, oral ulcers, and odynophagia associated with solids more than liquids. Due to the patient's symptoms, she was admitted to the local hospital's intensive care unit (ICU). During hospitalization, her dyspnea exaggerated to MMRC grade IV. Due to the requirement of ICU support, she was referred to a tertiary care institute in Uttarakhand on Day eight.

On examination, the patient was conscious. Her vital signs were as follows: Glasgow Coma Scale: E4V5M6, heart rate 112 beats per minute, oxygen saturation 60% in room air and 88% with 10-L oxygen facemask, blood pressure 108/78 mmHg, and respiratory rate 32 breaths per minute. Pallor and icterus were present on general examination. Local examination of the oral cavity revealed multiple yellowish plaques present over the tongue, buccal mucosa, and posterior wall of the pharynx. Respiratory system examination showed bilateral crepitations in the infraaxillary regions. Other system examinations were normal.

Laboratory investigations revealed anemia with neutrophilic leukocytosis, hyperbilirubinemia, transaminitis, elevated urea, and elevated creatinine, as shown in Table 1.

**Table 1:** Blood test results ALT, Alanine aminotransferase; AST, Aspartate aminotransferase; ALP, Alkaline phosphatase; GGT, Gamma Glutamyl Transpeptidase

Parameters	Results	Reference range
Hemoglobin	9.6 g/dL (5.96 mmol/L)	12-15 g/dL (7.45-9.31 mmol/L)
Red Blood Cell Count (RBC)	2.66 × 10^6^/mcL (2.66 × 10^12^/L)	3.8-5.2 × 10^6^/mcL (3.8-5.2 × 10^12^/L)
White Blood Cell Count(WBC)	16.09 × 10^3^/mcL (16.09 × 10^9^/L)	4-11 × 10^3^/mcL (4-11 × 10^9^/L)
Platelets	216 × 10^3^/mcL (216 × 10^9^/L)	150-400 × 10^3^/mcL (150-400 × 10^9^/L)
Total bilirubin	7.69 mg/dL (131.5 µmol/L)	0.3-1.2 mg/dL (5.13-20.52 µmol/L)
Direct bilirubin	5.66 mg/dL (96.79 µmol/L)	0-0.2 mg/dL (0-3.42 µmol/L)
Alanine aminotransferase (ALT)	77 U/L (1.28 µkat/L)	0-35 U/L (0-0.58 µkat/L)
Aspartate aminotransferase (AST)	83 U/L (1.38 µkat/L)	0-35 U/L (0-0.58 µkat/L)
Alkaline phosphatase (ALP)	562 U/L (9.33 µkat/L)	30-120 U/L (0.5-2 µkat/L)
Gamma Glutamyl Transpeptidase (GGT)	2943 U/L (49.06 µkat/L)	0-38 U/L (0-0.63 µkat/L)
Serum Albumin	3.13 g/dL (31.3 g/L)	3.5-5.2 g/dL (35-52 g/L)
Urea	181 mg/dL (30.13 mmol/L)	17-43 mg/dL (2.83-7.15 mmol/L)
Creatinine	6.16 mg/dL (544.67 µmol/L)	0.55-1.02mg/dL (48.63-90.19 µmol/L)
Sodium	138 mEq/L (138 mmol/L)	136-146 mEq/L (136-146 mmol/L)
Potassium	3.4 mEq/L (3.4 mmol/L)	3.5-5.1 mEq/L (3.5-5.1 mmol/L)
Calcium	8.1 mg/dL (2.02 mmol/L)	8.8-10.6 mg/dL (2.2-2.64 mmol/L)

Initial arterial blood gas (ABG) revealed respiratory alkalosis with adequate compensation, as shown in Table 2.

**Table 2:** Arterial Blood Gas results on different days ABG, Arterial Blood Gas

Arterial Blood Gas (ABG) parameters	At the time of admission	12 hours after admission	Day 2 of admission	Day 8 of admission	Reference range
pH	7.524	7.469	7.234	7.213	7.35-7.45
pCO2	26.2 mmHg	32.4 mmHg	54.2 mmHg	60.9 mmHg	35-45 mmHg
HCO3	21.8 mmol/L	23.7 mmol/L	28.6 mmol/L	24.1 mmol/L	22-26 mmol/L

**Figure 1:**
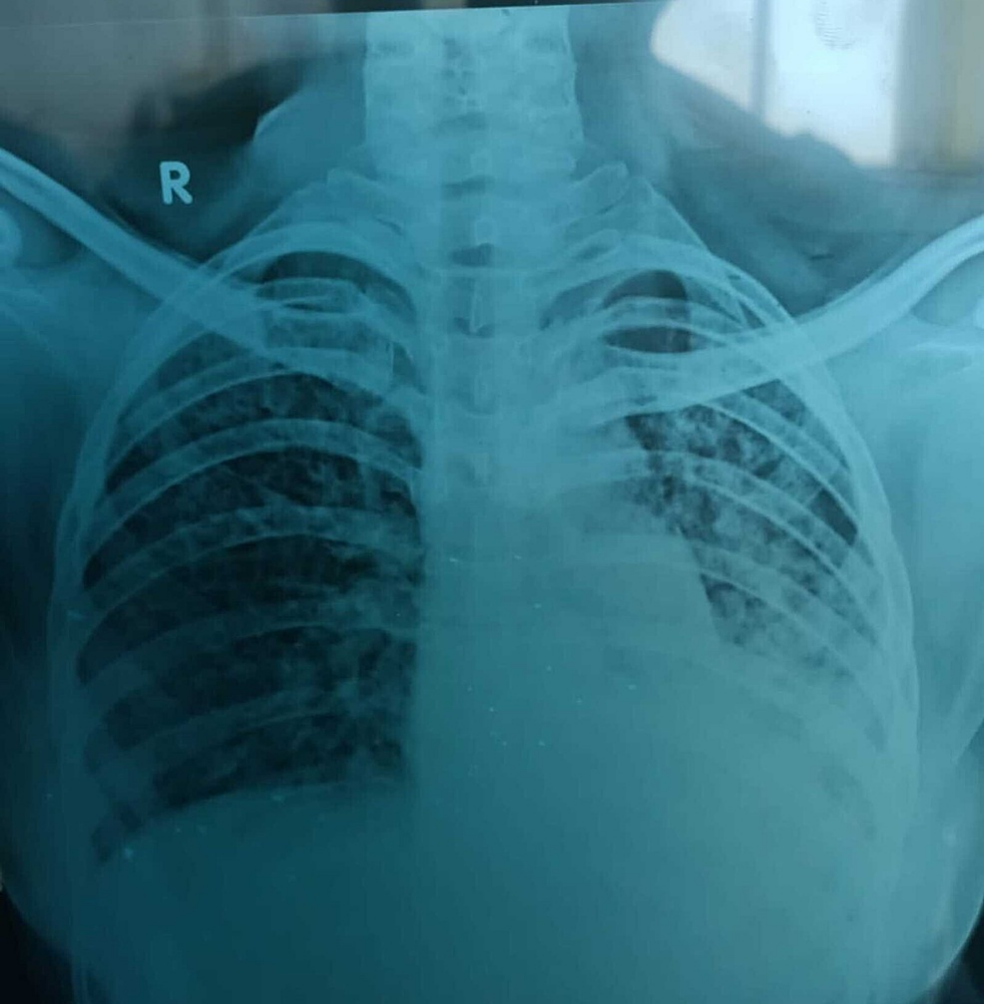
Chest radiograph on day one of admission Chest radiograph shows left lower lobe haziness, enhanced bronchovascular markings, and multiple patchy consolidations.

Chest radiograph revealed left lower lobe haziness, enhanced bronchovascular markings, and multiple patchy consolidations, as shown in Figure 1.

**Figure 2:**
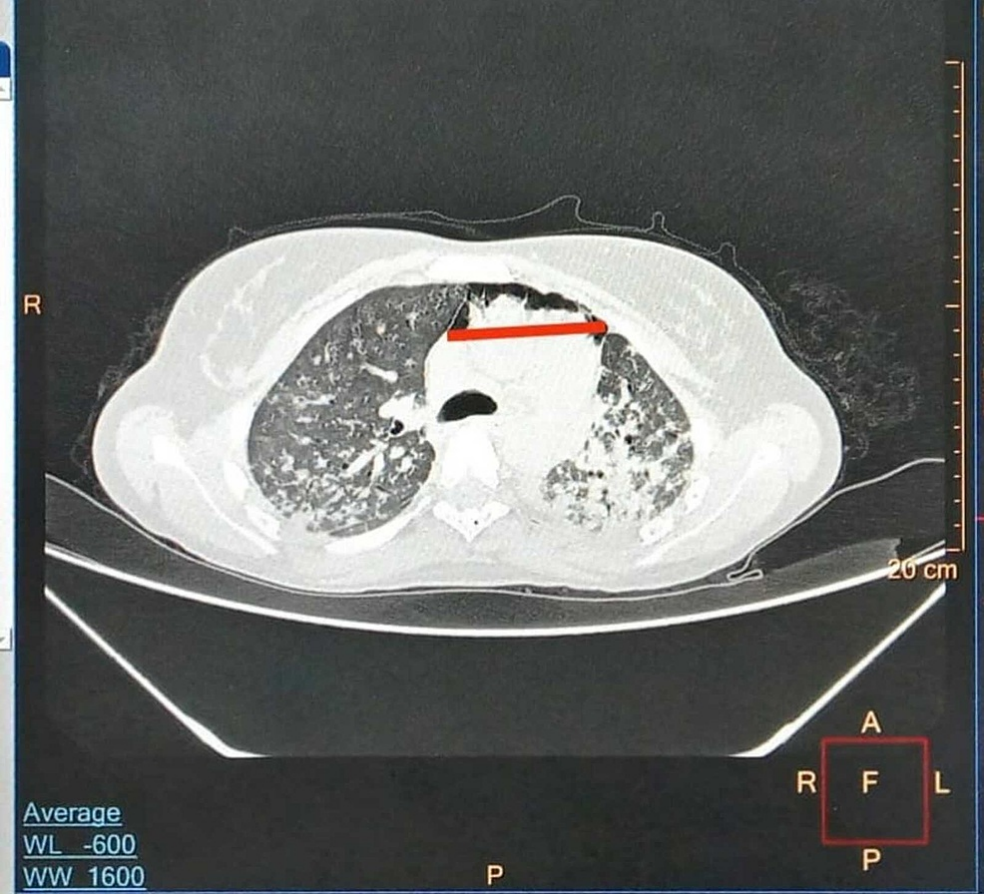
High-resolution computed tomography of the thorax High-resolution computed tomography image of the chest showing extensive ground glass opacity with pneumomediastinum of size 8.5 mm.

High-resolution computed tomography of the thorax revealed extensive ground-glass opacity with pneumomediastinum of size 8.5 mm (Figure 2).

**Figure 3:**
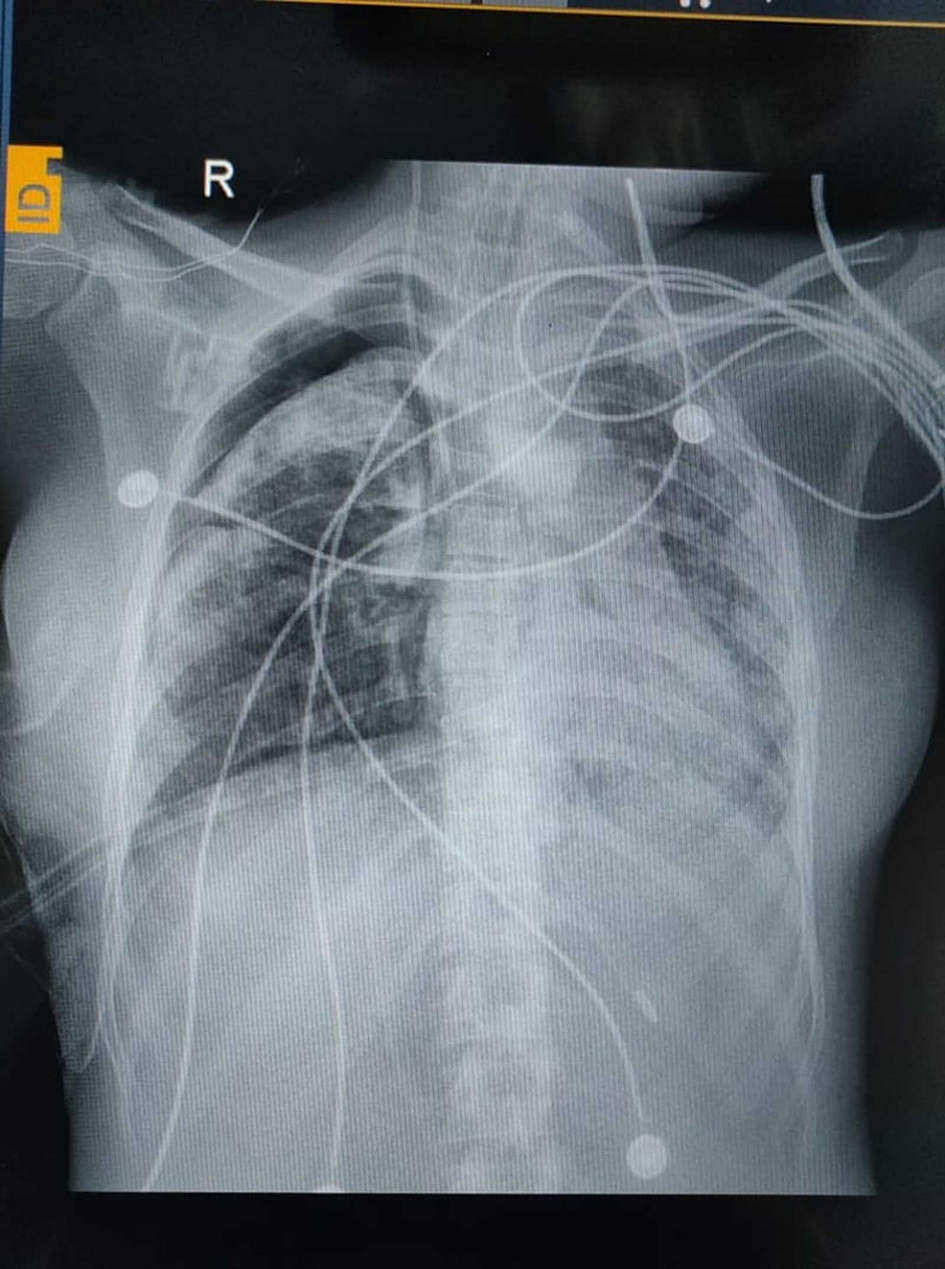
Chest radiograph on day five of admission Chest radiograph shows right-sided pneumothorax.

In the emergency room, the patient was started on ceftriaxone (1g twice daily), azithromycin (500 mg once daily), and dexamethasone (6 mg thrice daily) intravenously along with oxygen titration to maintain proper saturation (85%-88%). N-Acetyl cysteine (dose as in case of paracetamol overdose) was given because of existing acute liver injury and inotrope support was started due to worsening mean arterial pressure. On the second day of admission, she was placed on noninvasive ventilation due to worsening respiratory distress. On day five of admission, she developed a spontaneous pneumothorax (Figure 3), and a thoracostomy tube was placed.

On day eight, she was intubated because of increasing shortness of breath and placed on volume control mode. Procalcitonin elevated from 9.7 ng/mL (on day one) to 37.7 ng/mL (on day five); therefore, antibiotics were escalated to meropenem (500 mg twice daily). Despite all efforts, the patient expired on day 17 after admission (day 29 after ingestion of paraquat).

## Discussion

Paraquat was first synthesized in 1882 as a redox indicator, and later, its herbicidal properties were recognized in the 1950s [7]. The first case of paraquat-related systemic toxicity was reported in 1966 and is a common cause of herbicide poisoning in South-East Asia, with fatality rates around 50-90% [8,9]. Paraquat ingestion can be mild, moderate to severe, or fulminant or hyperacute with corresponding doses of less than 20-30 mg, more than 20-30 mg but less than 40-50 mg, and more than 40 to 55 mg paraquat ion per kilogram of body weight, respectively [7,10]. In our case, there was poisoning with a fulminant amount of paraquat poisoning.

In 1990, Daisley and Barton first described pneumothorax and pneumomediastinum induced by paraquat poisoning. The mechanism of Daisley Barton Syndrome is an initiation of pathological repair at the alveolar level with interstitial widening, congestion of the alveoli, deposition of collagen, and microthrombi formation leading to secondary pulmonary hypertension, a consequence of paraquat-induced pneumothorax and pneumomediastinum [6]. Damage to type 1 pneumocytes impairs oxygenation and capillary exchange, whereas type 2 pneumocyte damage leads to increased surface tension and fluid accumulation, causing pulmonary edema and hemorrhage [11,12]. Pneumomediastinum may be caused by corrosion and esophageal perforation due to the corrosive effect of paraquat or by air leakage from ruptured alveoli along with peribronchial vascular structures [2]. In our case, there was pneumomediastinum of size 8.5mm initially and later, pneumothorax.

Early management of paraquat poisoning is essential to prevent further absorption and gastric decontamination. We avoided gastric lavage due to the corrosive nature of the poison. Activated charcoal (1-2 g/kg) and Fuller’s earth (1-2g /kg) with 70% sorbitol are generally used for gastric decontamination [13]. However, there is no specific antidote for paraquat poisoning. Other treatment modalities include high dose vitamin C or E, N-acetyl cysteine, corticosteroids, cytotoxic agents like cyclophosphamide and azathioprine [7]. We used antibiotics, corticosteroids, and N-acetyl cysteine for treating the patient. Patients with paraquat poisoning will be harmed by supplemental oxygen due to free radical formation [9]. Therefore, it is best to avoid oxygen supplementation unless hypoxemic and target saturation is 85%-88%, as was done for treating our patient [14]. Hemodialysis and hemofiltration are uncommon, but allow a longer duration of continuous removal for two to five days [15]. A bedside semi-quantitative test using bicarbonate and sodium dithionite can be used to confirm systemic paraquat toxicity [9].

## Conclusions

Severe paraquat poisoning is associated with a poor prognosis, and there is no specific treatment associated with its ingestion. Pneumomediastinum is an early predictor of mortality in patients with paraquat poisoning, associated with 100% mortality, especially within the first eight days. Sodium dithionite can be used as a good predictor of outcome in paraquat poisoning. Additionally, oxygen should be used only if hypoxemic. Early initiation of treatment and retarding the progress of poisoning offers the only chance of survival in patients with paraquat poisoning.
